# Effects of a physical activity program in stress management and motivation for the regular practice of physical activity of teachers from Portugal and Brazil

**DOI:** 10.1192/j.eurpsy.2023.2094

**Published:** 2023-07-19

**Authors:** A. P. Amaral, L. Mariano, H. Simões, M. Pocinho

**Affiliations:** 1 Coimbra Health School, Polytechnic of Coimbra; 2 Polytechnic of Coimbra, Coimbra, Portugal

## Abstract

**Introduction:**

Teaching is considered by the International Labor Organization as one of the most stressful professions, with consequences on the mental and physical health and on their professional performance. Intervention programs focused on physical activity usually present a significant decrease in the level of stress and an improvement in the quality of life of teachers. Physical activity is considered one of the main non-pharmacological strategies to reduce stress, generating a positive influence on mental health.

**Objectives:**

To evaluate the impact of an intervention program based on the practice of physical activity on the level of stress and motivation for the regular practice of physical activity in teachers.

**Methods:**

This study employed a pretest-posttest design. Measures: Portuguese versions of Stress Perception Scale and Motivation Inventory for Regular Physical Activity Practice. Sample: 33 teachers from Portugal and Brazil, 57,6% females, 54,5% from Portugal, 63,6% with age between 26 and 35 years. The intervention ran for 8 weeks, with a total of 40 sessions with cardiorespiratory conditioning exercises, muscle strengthening and stretching, relaxation and meditation techniques.

**Results:**

On baseline we found significant relationship between “years of teaching experience” and the level of stress (p=.027). After the intervention, the level of stress significantly decreased in Portuguese teachers (p=.031). In 83% of the sample, there is a decrease in the levels of perceived stress. Concerning Brazilian teachers (p=.006), in 73% of the sample, there is a decrease in the levels of perceived stress. Regarding motivation, there is a significant increase in Portuguese teachers related to “stress control” (p<.001), “sociability” (p=.001), “competitiveness” (p<.001), and “esthetic” (p=.004). In Brazilian teachers there is an increase related to “stress control” (p=.003), and “competitiveness” (p=.001).

**Conclusions:**

Both samples showed positive results, attesting the efficacy of the intervention based in physical exercises to reduce stress and increase motivation to practice physical activity regularly. After intervention, more motivational dimensions are changed in Portuguese teachers, comparing with Brazilian teachers.

**Disclosure of Interest:**

None Declared

